# Co-dependent regulation of p-BRAF and potassium channel KCNMA1 levels drives glioma progression

**DOI:** 10.1007/s00018-023-04708-9

**Published:** 2023-02-10

**Authors:** Shanshan Xie, Chengyan Xu, Cheng Wu, Yuhan Lou, Jingwei Duan, Rong Sang, Ziwei Lou, Jiaru Hou, Wanzhong Ge, Yongmei Xi, Xiaohang Yang

**Affiliations:** 1 The Women’s Hospital, Institute of Genetics, Zhejiang Provincial Key Laboratory of Genetic and Development Disorders, School of Medicine, Zhejiang University, Hangzhou, 310058 China; 2 Department of Neurosurgery, The Children’s Hospital Zhejiang University School of Medicine, National Clinical Research Center for Child Health, Hangzhou, Zhejiang China; 3 Joint Institute of Genetics and Genomic Medicine Between Zhejiang University and the University of Toronto, Zhejiang University, Hangzhou, 310058 China

**Keywords:** BRAF, KCNMA1, Glioma, Potassium channel

## Abstract

*BRAF* mutations have been found in gliomas which exhibit abnormal electrophysiological activities, implying their potential links with the ion channel functions. In this study, we identified the *Drosophila* potassium channel, Slowpoke (Slo), the ortholog of human KCNMA1, as a critical factor involved in *dRaf*^*GOF*^ glioma progression. Slo was upregulated in *dRaf*^*GOF*^ glioma. Knockdown of *slo* led to decreases in dRaf^GOF^ levels, glioma cell proliferation, and tumor-related phenotypes. Overexpression of *slo* in glial cells elevated dRaf expression and promoted cell proliferation. Similar mutual regulations of p-BRAF and KCNMA1 levels were then recapitulated in human glioma cells with the *BRAF* mutation. Elevated p-BRAF and KCNMA1 were also observed in HEK293T cells upon the treatment of 20 mM KCl, which causes membrane depolarization. Knockdown *KCNMA1* in these cells led to a further decrease in cell viability. Based on these results, we conclude that the levels of p-BRAF and KCNMA1 are co-dependent and mutually regulated. We propose that, in depolarized glioma cells with *BRAF* mutations, high KCNMA1 levels act to repolarize membrane potential and facilitate cell growth. Our study provides a new strategy to antagonize the progression of gliomas as induced by BRAF mutations.

**Supplementary Information:**

The online version contains supplementary material available at 10.1007/s00018-023-04708-9.

## Introduction

BRAF is a serine/threonine protein kinase in the RAS/RAF/MEK pathway. It plays an important role in cell proliferation and differentiation [1, 2]. Abnormalities in the proto-oncogene *BRAF* have been found in various tumors [2–5].* BRAF*^*V600E*^, the typical oncogenic mutation, accounts for about 90% of *BRAF* mutation tumors [6, 7]. In the central nervous system, *BRAF* mutations are usually found in gliomas, such as the pilocytic astrocytomas, diffuse midline gliomas, and in high grade astrocytomas with piloid features [8]. In children, pediatric low-grade gliomas (pLGGs) also exhibit high rates of *BRAF* alteration. *BRAF*^*V600E*^, for example, was found in about 10–20% of all pLGGs [9–13]. Patients with *BRAF*^*V600E*^ usually exhibited worse outcomes than those with non-mutated *BRAF* pLGG [13]. Mechanistically, *BRAF* gain-of-function mutations [14, 15], or the activation of the MAPK pathway [16–18], clearly altered the electrophysiological properties of neurons. Brain slices carrying the *BRAF*^*V600E*^ mutation showed significant increases of focally spontaneous firing rates, suggesting a relationship between *BRAF*^*V600E*^ mutation and seizures [19]. When presented in astrocytes, *BRAF*^*V600E*^ also led to hyper-excitable intrinsic properties such as threefold increases in action potential firing rates, low thresholds for the firing of action potentials, depolarized membrane potentials, and elevated hyperpolarization-activated inward currents [20]. Though ion-channel dysfunction was strongly implicated in such observations, how ion channels specifically function in *BRAF* glioma progression currently remained unclear.

*KCNMA1* encodes a pore-forming α-subunit of the large-conductance Ca^2+^-activated K^+^ channel [21]. KCNMA1 mediates the out-flow of K^+^ and is regulated by membrane depolarization and intracellular levels of Ca^2+^ [22, 23]. It plays multi-effect roles in many physiological processes, including membrane potential repolarization [24] and in the control of nerve excitability and neurotransmitter release [25]. Accumulating evidence suggests that K^+^ channels may be involved in tumorigenesis in a variety of tissues [26–31]. Differential expression levels of *KCNMA1* in different cancer cells may reflect multiple-related functions in tumor growth. *slowpoke* (*slo*) is the ortholog of human *KCNMA1* in *Drosophila* [32–34]. It is widely expressed throughout excitable tissues where it affects action potential shape, neural excitability, transmitter release, and smooth muscle tone [35]. Studies of *slo* mutants have revealed the corresponding roles of K^+^ channels in neuronal functions, circadian activity [34, 36].

The *Drosophila* larval brain is a unique system for genetic screening identifying factors that inhibit tumor growth. *Drosophila Raf* (*dRaf*) is the only ortholog of human *BRAF*. Overexpression of a truncated form of dRaf (dRaf^GOF^) in glial cells of the larval brains led to glioma phenotypes such as enlarged brain sizes, over-proliferation of glial cells, seizure-like behavior in adult animals, and shortened lifespans [37, 38].

In this study, we employed a *Drosophila Raf*^*GOF*^ glioma model and identified that Slo was required for tumor progression. dRaf^GOF^ and Slo expression levels were revealed as co-dependent and mutually regulated in glioma brains. This co-dependency was recapitulated in human cells where elevated KCNMA1 was observed in gliomas with *BRAF*^*GOF*^ mutation. In such case knockdown of *KCNMA1* reduced p-BRAF levels and inhibited cell growth. Our data suggests that this co-dependent regulation of p-BRAF and KCNMA1 levels is mediated by a membrane potential-sensitive mechanism. Our study may lead to KCNMA1 becoming highlighted as a potential drug target that could be functionally-specific for tumors induced by *BRAF* mutations.

## Materials and methods

### Fly stocks and genetics

All flies were kept at 25 °C with standard cornmeal/yeast/agar medium. Late 3rd instar larvae were collected for studies unless otherwise stated. Sources of *Drosophila* stocks in this study are listed below: w^*1118*^ (THJ025, Tsinghua Drosophila center), *repo*-GAL4, UAS-eGFP/CyO; UAS-*Raf*^*GOF*^/TM6B GAL80 (gifts from Xi Huang’s lab), *slo* RNAi (THU2519.N and THU2246, Tsinghua Drosophila center), *slo*^*KO*^ (Generated in this study), *slo* gRNA (79777, Bloomington), *nos*-GAL4, UAS-Cas9 (67083, Bloomington), UAS-*slo* (Generated in this study).

### Fly seizure-like behavior test

Analysis of seizure phenotype induced by heat-shock was performed in transparent tubes with 20 flies in each tube. Flies were heat shocked at 37 °C for the given time up to 10 min. Within 10 min after treatment, each occasion a fly dropped from the tube wall and lay upside-down at the bottom was counted as one seizure-like event.

### Samples from patients

Five samples were obtained following informed consent from patients. All experimental procedures were performed in accordance with the Research Ethics Board at the Children’s Hospital, Zhejiang University School of Medicine. The permit to use the clinical data was approved by the institutional review boards.

### Cell culture

DBTRG-05MG was purchased from the American Type Culture Collection (ATCC). T98G and U87-MG (from ATCC), were the gifts from Dr. Du Linyong. The HEK293T cell line (from ATCC) was a gift from Dr. Liang Hongqing. All cell lines were cultured in DMEM (GIBCO) at 37 °C in the presence of 5% CO_2_ with 10% fetal bovine serum (GIBCO) and 1% penicillin/streptomycin (Thermo Scientific, 100 U/ml penicillin, 100 µg/ml streptomycin). All lines were routinely checked for STR identification and mycoplasma contamination.

### Lentivirus production and infection

Lentiviral particles were generated by transfecting HEK293T cells with pLKO.1 -shRNA-copGFP-2A-PURO, pSAX2 and pVSVG at a ratio of 3:4:1. shRNAs in pLKO.1 were constructed commercially by the Tsingke company. The sequences of shRNA were as follows: NC-shRNA 5ʹ-CGTGACACGTTCGGAGAACC-3ʹ, *KCNMA1*-shRNA1 5ʹ -TTAACTCTATGATTTCAGGG-3ʹ, *KCNMA1*-shRNA2 5ʹ-TCTTTCCAATTCCAGCTCGG-3ʹ. Transfection was performed using Lipo8000™ Transfection Reagent (Beyotime) according to the manufacturer’s protocol. Virus-containing supernatant was collected and filtered (0.45 µm) at 48 h and 72 h after transfection. The viral supernatant was then used to transfect cells in the presence of polybrene (8 µg/ml) for 24 h and selected with puromycin (4 µg/ml) for 72 h.

### Antibody generation

*Slo* cDNA encoding aa 636 to 816 was cloned into a pGEX-4T-1 expression vector. The antigen was bacterially produced, and the antibody raised in rabbits according to the published protocol [39].

The same method was used to make the dRaf antigen (aa 285–421). Anti-dRaf was generated in guinea pigs.

### Antibodies used in this study

The commercial primary antibodies in this paper were as follows: anti-REPO (8D12, DSHB); anti-pH3 (Ser10) (H0412, Millipore); anti-Cleaved-Caspase 3 (9664, Cell Signaling); anti-α tubulin (ab18251, abcam); anti-MEK (8727, Cell Signaling); anti-p-MEK (Ser217/221) (3958, Cell Signaling); anti-ERK (sc-514302, Santa Cruz); anti-p-ERK (T202/Y264) (4370, Cell Signaling); anti-KCNMA1 (sc-374142, Santa Cruz); anti-Ki-67 (9129, Cell Signaling); and anti-GAPDH (ab8245, abcam).

Three BRAF-related antibodies were used in this study: anti-BRAF (sc-5284, Santa Cruz) were used to detect the total levels of BRAF; anti-BRAF^V600E^ specifically recognizing mutated amino acid sequence (ab228461, abcam) were used to detect the levels of BRAF^V600E^; anti-p-BRAF (S729) specifically recognizing phosphorylated site (ab124794, abcam) were used to detect the levels of BRAF with serine 729 phosphorylation, to measure the BRAF activation levels regardless of the presence of BRAF mutations.

### Immunofluorescence staining

For brain immunostaining, late 3rd instar larval brains were dissected in ice-cold PBS (10 mM NaH2PO4/Na2HPO4, 175 mM NaCl, pH7.4) and fixed for 15 min in PBS with 4% paraformaldehyde at room temperature. For cell immunostaining, cells on slides were fixed for 15 min in PBS with 4% paraformaldehyde at room temperature. All samples were incubated with primary antibodies at 4 °C overnight, followed by washes and mixture of the secondary antibodies at room temperature for 2 h [39, 40]. All commercial secondary antibodies used were from the Jackson Laboratory. DNA was stained with DAPI (C1002, Beyotime) at 1:2000. Antifade Mounting Medium (P0126, Beyotime) was used to protect the fluorescent signals. Images were obtained using an Olympus FV1000 confocal microscope and processed with Adobe Photoshop.

### Quantification of mitotic glial cells

All samples were immunofluorescent stained with primary antibody (anti-Repo, anti-PH3), and appropriate secondary antibodies. The proliferating glial cells were double-labelled by anti-Repo and anti-PH3. Mitotic glial cells were quantified by counting the total numbers of proliferating glial cells in a hemispheric lobe.

### Western blot

Late 3rd instar larval brains or cultured cells were lysed in RIPA lysis buffer [50 mM Tris–HCl pH 8.0, 150 mM NaCl, 0.5% sodium deoxycholate, 0.1% SDS, 1% IGEPAL CA‐630 and complete protease inhibitor cocktail (Roche)]. Samples were subjected to SDS-PAGE and transferred to a polyvinylidene fluoride membrane (Millipore #IPVH0001). Western blot assays were performed according to the published protocol [39]. Immunoreactive bands were visualized using Clinx ChemiScope.

### Immunohistochemistry of tissue sections

Anti-KCNMA1 was used to stain the paraffin embedded sample sections performed by the Core Facilities, Zhejiang University, School of Medicine. Images were taken using a Nikon ECLIPSE 80*i* microscope*.*

### Quantitative RT-PCR

RNA was extracted using the TRIzol (Invitrogen) method. A cDNA Synthesis Kit (Vazyme) was then used according to the manufacturer's protocol. A 7900HT fast real-time PCR system (Applied Biosystems) was used for quantitative RT-PCR. PCR was carried out using a Power SYBR Green PCR Mix Kit (Vazyme) in 96-well plates following published methods [39]. *rp49* was used as an internal control for *Drosophila* brain samples and *GAPDH* for human samples. The primer for RT-PCR in this study: for *slo*, forward 5ʹ-GCCCATGATAACAGAACTGG-3ʹ, reverse 5ʹ-GTAAGTCGTGGACATCAGC-3ʹ; for *Raf*, forward 5ʹ-GAGGAAAACTGGAATATTCTGGCG-3ʹ, reverse 5ʹ-CTCGGTGTCTTCACGTTGAGTGTC-3ʹ; for *rp49*, forward 5ʹ-GCTAAGCTGTCGCACAAA-3ʹ, reverse 5’-TCCGGTGGGCAGCATGTG-3ʹ; for *BRAF*, forward 5ʹ-TACCTGGCTCACTAACTAACGTG-3ʹ, reverse 5ʹ-CACATGTCGTGTTTTCCTGAG-3ʹ; for *KCNMA1*, forward 5ʹ-GGCAGCAGTCTTAGAATGAGTAG-3ʹ, reverse 5ʹ-AAAGCCCACCACATGCGTT-3ʹ; for *GAPDH*, forward 5ʹ-CTCCTGCACCACCAACTGCT-3ʹ, reverse 5ʹ-GGGCCATCCACAGTCTTCTG-3ʹ.

### Assessment of cell proliferation by CCK8

Cells were seeded (5000/well) and maintained in 96-well plates. For the determination of cell proliferation, a Cell Counting Kit-8 (CCK8; Glpbio.GK10001) was used according to the user’s manual. Fluorescence (450 nm) was recorded on a microplate reader (Biotek Synergy H1).

### Statistical analysis

All data are expressed as the mean ± SD. All statistical data are processed using unpaired two-tailed Student’s *t* test in GraphPad Prism.

## Results

### Slo knockdown inhibits* Drosophila Raf*^*GOF*^ glioma symptoms

**Fig. 1:**
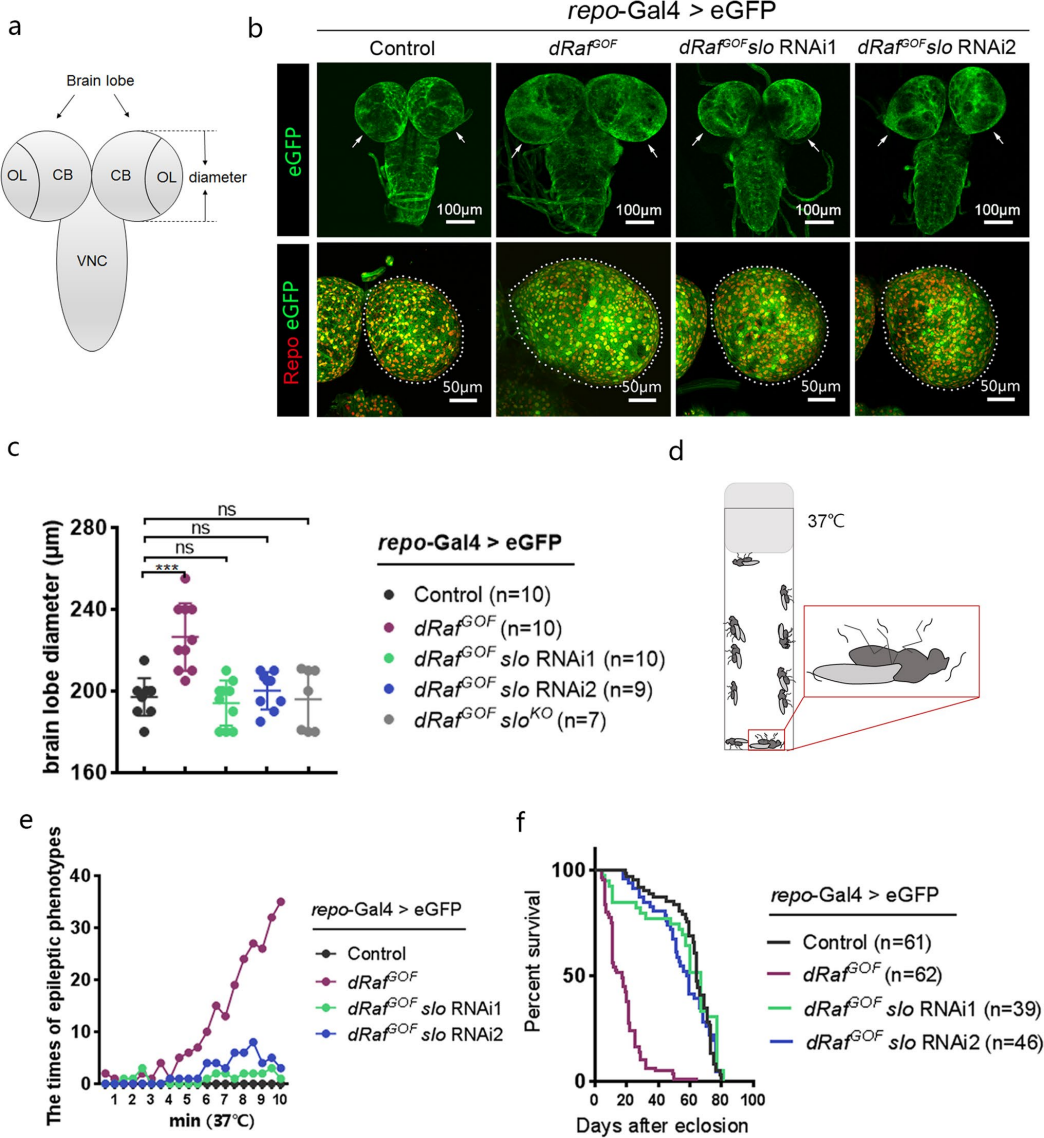
Lack of Slo inhibits *Drosophila Raf*^*GOF*^ glioma growth and rescues the glioma phenotype. **a** Schematic diagram of the *Drosophila* larval central nervous system including brain lobes (arrowhead) and ventral nerve cord (VNC). The brain lobe contains the central brain (CB) and optic lobe (OL) regions. Measured diameters of larval brain lobes are indicated by two-dotted lines. **b** Confocal images of the third-instar larval brains of the control, *dRaf*^*GOF*^ glioma, and *dRaf*^*GOF*^ glioma with *slo*-RNAi treatments (anti-Repo, red and eGFP, green). The enlarged brain lobes (arrow) of *dRaf*^*GOF*^ gliomas were rescued by either of the two *slo*-RNAi as driven by *repo*-Gal4. **c** Statistical analysis of the diameter of the third-instar larval brain lobes of the control, *dRaf*^*GOF*^ glioma, *dRaf*^*GOF*^ glioma with *slo*-RNAi treatments, and *dRaf*^*GOF*^ glioma with *slo*^*KO*^. The data are plotted as mean ± SD. ***P < 0.001. ns, not significant. **d** Schematic presentation of *Drosophila* epileptic phenotype induced by heat shock. **e** Frequencies of epileptic behaviors of adult flies induced by various heat shock time at 37C. *n* = 20. **f** Lifespan of the adult flies of the control, *dRaf*^*GOF*^ glioma, and two *dRaf*^*GOF*^ glioma with *slo*-RNAi treatments

To explore the potential roles of ion channels in tumor growth, we employed a *Drosophila* glioma model induced by constitutively activated *Raf* (*dRaf*^*GOF*^) in the glial cells of larval brains. This model mimicked gliomas in patients with aberrant activations of BRAF [38, 41], with enlarged brain lobe sizes as marked by eGFP driven by the glial specific *repo*-GAL4 (Fig. 1b). We performed knockdown screens of ion channel genes with RNAi lines available, and quantified the brain lobe diameters of the 3rd instar larvae as the primary parameter to evaluate the functions of ion channels on tumor growth. The *Drosophila* line with *repo*-GAL4, UAS-eGFP only was used as control in this study. Ameliorative phenotypes were observed for *dRaf*^*GOF*^ glioma brains with *slo* knockdown (Fig. 1a, b). Two independent *slo*-RNAi lines targeting non-overlapping regions of *slo* transcript (RNAi1, THU2519.N and RNAi2, THU2246 from Tsinghua *Drosophila* Center) were crossed into the *dRaf*^*GOF*^ glioma background, respectively. Glial cell-specific knockdown of *slo* in *dRaf*^*GOF*^ gliomas completely prevented brain sizes (Fig. 1a–c).

To eliminate the possibility of off-target effects in RNAi experiments, a *slo* genetic mutation was generated using the CRISPR/Cas9 method. The mutant harbored a frame shift mutation with a 2 bp deletion in codon 85, resulting in a truncated protein (Fig. S1a and S1b). We named this *slo* mutation as *slo*^*KO*^. Homozygous *slo*^*KO*^ mutant flies were viable and produced progeny, albeit with a weak locomotion defects as reported earlier [42, 43]. When the homozygous *slo*^*KO*^ was introduced into the *dRaf*^*GOF*^ background, the brain lobe sizes of the 3rd instar larvae were significant smaller than those without such a *slo*^*KO*^ introduction. This was consistent with the results from RNAi experiments (Fig. S1c, Fig. 1c). This observation confirms that the *slo* RNAi rescue phenotype is not an off-target event. Interestingly, neither knocking down *slo* in glial cells of *wt* brains nor the homozygous *slo*^*KO*^ animals resulted in any discernible effects on their brain sizes or total numbers of mitotic glial cells as compared with *wt* brains (Fig. S2a–S2c). The viabilities of *slo* RNAi groups were not significantly altered, neither (Fig. S2d). These findings suggest that, although Slo plays a very prominent role in *dRaf*^*GOF*^-induced glioma growth, its functions are largely dispensable for normal glial cell proliferation.

Recent studies have shown that the low-grade gliomas with *BRAF* mutations have been frequently accompanied by epileptic seizures [19, 44, 45]. Similar spontaneous epileptic phenotype was detected and adult animals lying upside-down were observed in *dRaf*^*GOF*^ glioma flies (Fig. 1d). A quick and well-established method to induce the seizure-like behavior in adult flies is to heat-shock the animals at 37 °C [46–49]. However, the *dRaf*^*GOF*^ glioma flies exhibited seizures at a greatly reduced heat shock time as compared to control flies. Four and half minute heat-shock began inducing weak but reproducible seizure-like activities in *dRaf*^*GOF*^ glioma flies (Fig. 1e), and the phenotype continued to be greatly enhanced from this point on. No *wt* flies showed any corresponding seizures (Fig. 1e). Such seizures were largely avoided by *slo* RNAi treatments (Fig. 1e). We also performed Kaplan–Meier survival curve analyses. Since male flies showed more pronounced survival rate changes, only male flies were used in this study. Results showed a significantly shorter median survival for *dRaf*^*GOF*^ glioma group (17 days, *P* < 0.0001) as compared with the control group (64 days); while *dRaf*^*GOF*^ glioma with *slo* RNAi groups showing extended median survival to 67 days and 58.5 days, respectively (Fig. 1f).

### Elevated Slo expression promotes *dRaf*^*GOF*^ glioma cell proliferation

**Fig. 2:**
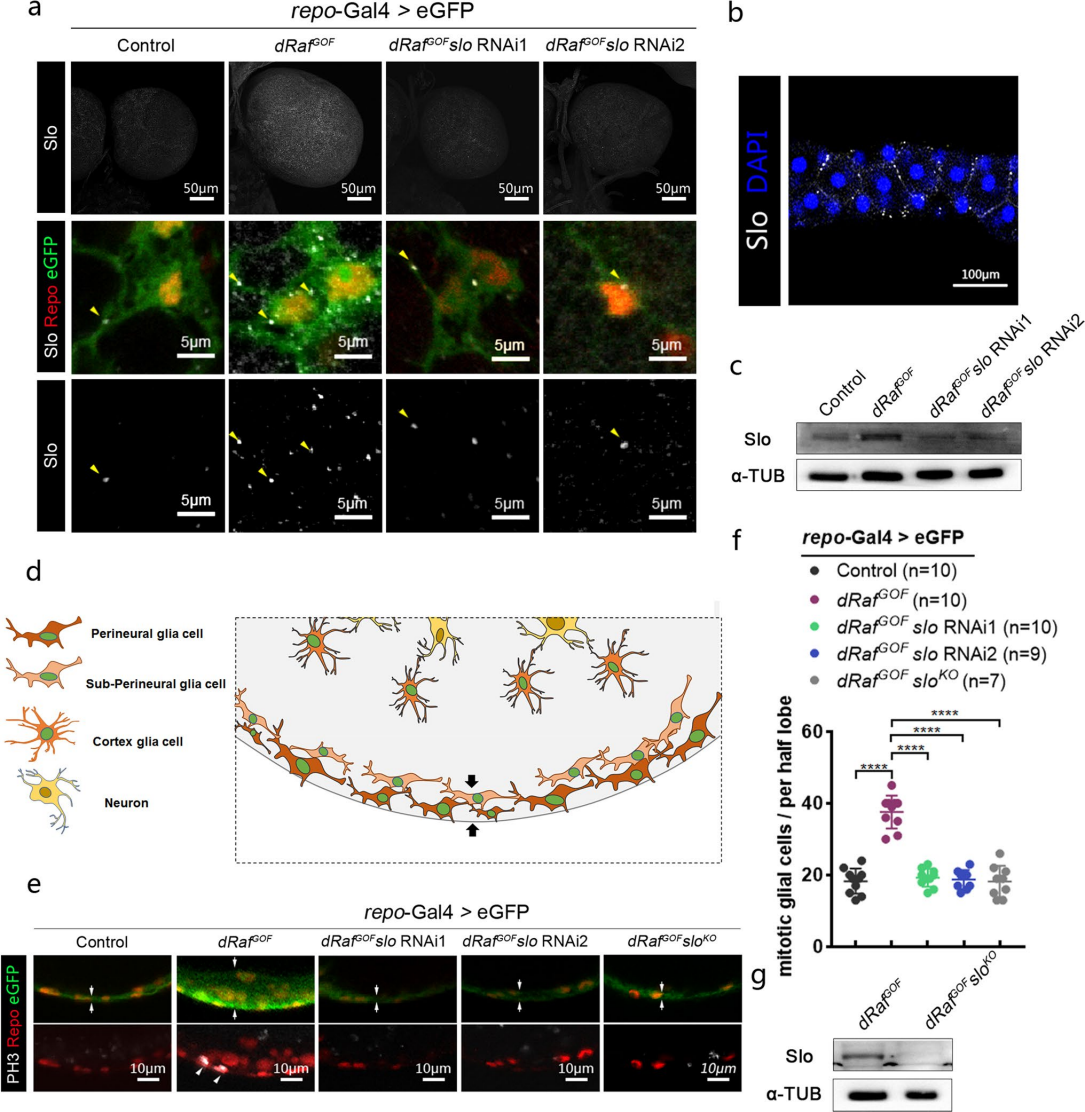
Membrane-bound protein Slo is highly expressed in *dRaf*^*GOF*^ glioma and associated with cell proliferation. **a** Confocal images of the third-instar larval brains of the control, *dRaf*^*GOF*^ glioma, and *dRaf*^*GOF*^ glioma with *slo*-RNAi treatments (anti-Slo, white; anti-Repo, red and eGFP, green). Arrowheads point to the Slo positive punctate signals. Increased numbers of Slo positive punctate signals were found in *dRaf*^*GOF*^ gliomas and such signals were decreased with *slo-*RNAi treatments. **b** Confocal images of larval salivary gland labeled by anti-Slo (white) and DAPI (blue). **c** Western blot results showing protein levels of Slo as elevated in *dRaf*^*GOF*^ gliomas as compared to controls and that such elevated protein levels were prevented by *slo*-RNAi treatment. α-tubulin (α-TUB) was used as internal reference. **d** Cross section diagram of three glial cell types in third-instar larval brains. Perineural and sub-perineural glial cells function as the brain blood barrier (BBB, between two arrows). **e** Confocal images of the BBB in third-instar larval brains lobes of the control, *dRaf*^*GOF*^ glioma, *dRaf*^*GOF*^ glioma with *slo*-RNAi treatments and *dRaf*^*GOF*^ glioma with *slo*^KO^ (anti-Repo, red; eGFP, green; anti-PH3, white). The distances between two arrows indicate the thickness of the BBB. The arrowheads show the mitotic glia cells double labeled by anti-Repo (red) and anti-PH3 (white). **f** Statistical analysis of the mitotic glia cells for each half brain lobe of the control, *dRaf*^*GOF*^ glioma, *dRaf*^*GOF*^ glioma with *slo*-RNAi treatments and *dRaf*^*GOF*^ glioma with *slo*^*KO*^. The data are plotted as mean ± SD. ****P < 0.0001. **g** Western blot results showing protein levels of Slo in *dRaf*^*GOF*^ glioma and *dRaf*^*GOF*^ glioma with *slo*^*KO*^. α-tubulin (α-TUB) was used as internal reference

*slo* encodes a pore-forming K^+^ channel protein involved in electrophysiological processes. An antibody against the fragment of Slo from aa 636 to 816 was generated in rabbits and exhibited antigen specificity against Slo protein. Anti-Slo immunofluorescence staining showed punctate signals in glial cells of *Drosophila* 3rd instar larval brains (arrowheads, Fig. 2a), which was consistent with previous reports [50, 51]. In order to verify Slo membrane locations, we stained cells in the larval salivary glands where the Slo punctate signals were associated with cell membranes (Fig. 2b). The Slo signals were notably denser in *dRaf*^*GOF*^ gliomas, suggesting a higher Slo protein levels in tumor cells. Upon *slo* knockdown, punctate Slo signals were decreased significantly (arrowheads, Fig. 2a). The Western blot results clearly showed that the expression of Slo in *dRaf*^*GOF*^ glioma brains was significantly higher than that in the controls (Fig. 2c), while *slo* knockdown resulted in a reduction of Slo expression. These results were consistent with the immunofluorescence staining data (Fig. 2a).

The superficial layer of the larval brain lobes mainly contains three glial cell types: namely perineural, sub-perineural and cortex glial cells. Perineural, sub-perineural glial cells collectively function as the brain blood barrier (BBB, Fig. 2d) [52, 53]. Since the BBB is an easily accessible structure in *Drosophila* larval brains, we examined the glial cells in the BBB. In Confocal cross section images, notably thickened glial cell layers were observed in *dRaf*^*GOF*^ glioma brains as compared with those of the *wt* (between two arrows, Fig. 2d, e). When *slo* expression was knocked down with RNAi, the thickness of glial cell layers was then restored (Fig. 2e). We assumed that the discrepancy of glial layer thickness among different samples was due to the hyper-proliferation of glioma cells [54–56]. Therefore, a mitosis marker, anti-PH3, was used to visualize proliferating cells. Indeed, the total numbers of proliferating glial cells double-labeled by anti-PH3 and anti-Repo were almost doubled in *dRaf*^*GOF*^ glioma brains as compared with *wt* (Fig. 2e, f). However, knockdown of *slo* resulted in decreased numbers of these mitotic glial cells. These experiments were repeated with the *slo*^*KO*^ line and similar results were obtained (Fig. S3, Fig. 2e–g).

To exclude the possibility that apoptosis may contribute to the decrease in total glial cell populations in the *slo* RNAi experiments, we stained the brains with anti-Cleaved Caspase3 to evaluate the potential apoptosis in *slo* knockdown *dRaf*^*GOF*^ glioma brains. No apoptotic signal was detected in the control or glioma brains, or in the glioma brains with *slo* knockdown (Fig. S4), suggesting that the inhibition of glioma progression by *slo* knockdown was independent of apoptosis. Based on these data, we conclude that the elevated levels of Slo in glial cells promotes tumor progression in *dRaf*^*GOF*^ glioma brains.

### Expression of Slo and dRaf^GOF^are co-dependent and mutually regulated

**Fig. 3:**
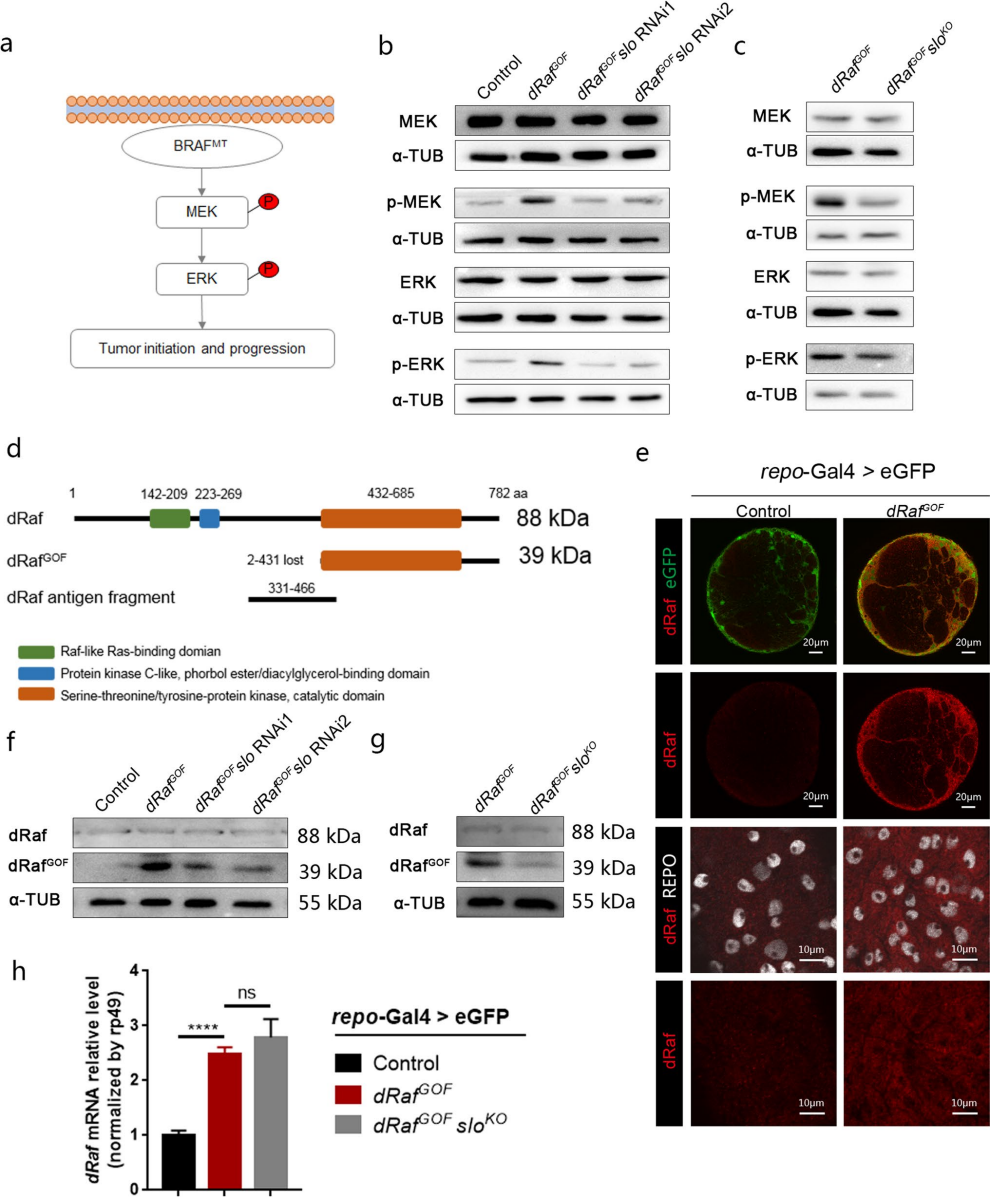
Slo and dRaf^GOF^ levels regulate MAPK pathway in *dRaf*^*GOF*^ glioma. **a** Diagram of potential tumorigenesis of BRAF mutation. **b** Western blot analysis of the control, *dRaf*^*GOF*^ gliomas, and *dRaf*^*GOF*^ gliomas with *slo*-RNAi treatments, showing the levels of proteins in the BRAF/MAPK pathway. Total levels of MEK and ERK remained unchanged, but p-MEK and p-ERK levels had increased significantly in *dRaf*^*GOF*^ gliomas. The elevated p-MEK and p-ERK were suppressed by *slo*-RNAi treatments. **c** Western blot analysis of *dRaf*^*GOF*^ gliomas and *dRaf*^*GOF*^ glioma with *slo*^*KO*^ also showed the same phenomenon in the BRAF/MAPK pathway. **d** Schematic presentations of the full-length dRaf (dRaf), truncated gain of function dRaf (dRaf^GOF^), and dRaf fragment used for antigen. **e** Confocal images of the labeled third-instar larval brain lobes of the control and *dRaf*^*GOF*^ glioma (eGFP, green, anti-dRaf, red and anti-Repo, white). **f** Western blot analysis of dRaf and dRaf^GOF^ levels of the control, *dRaf*^*GOF*^ glioma and *dRaf*^*GOF*^ glioma with *slo*-RNAi treatments. Full-length dRaf levels did not change while dRaf^GOF^ levels decreased upon *slo*-RNAi treatments. **g** Western blot analysis of dRaf and dRaf^GOF^ levels of *dRaf*^*GOF*^ glioma and *dRaf*^*GOF*^ glioma with *slo*^*KO*^. **h** Quantitative RT-PCR showing that total *dRaf* and *dRaf*^*GOF*^ mRNA levels in glioma is up-regulated compared with control, and had remained unchanged with or without *slo*^*KO*^. *rp49* as internal reference. ****P < 0.0001, ns, not significant. α-tubulin (α-TUB) was used as internal reference for all Western blot analyses

It is known that oncogenic *BRAF* mutations activate downstream MEK (MEK1 and MEK2) and ERK (ERK1 and ERK2) kinases in the BRAF/MAPK pathway (Fig. 3a) [1, 2]. To further probe the relationship between Slo and the BRAF/MAPK pathway, the expression levels of these proteins were examined. Western blot results showed that the total protein levels of both MEK and ERK remained unchanged, while levels of phosphorylated MEK (p-MEK) and phosphorylated ERK (p-ERK) were upregulated significantly in *dRaf*^*GOF*^ gliomas (Fig. 3b). This suggested that the BRAF/MAPK pathway was hyper-active. However, upon *slo-*RNAi treatments the activity of the BRAF/MAPK pathway was suppressed, as manifested by the reduced levels of p-MEK and p-ERK (Fig. 3b). This experiment was repeated with the *slo*^*KO*^ line and similar results were observed (Fig. 3c). This demonstrates that elevated Slo expression is required for BRAF/MAPK pathway activation in *dRaf*^*GOF*^ gliomas and that the knockdown of *slo* reduces p-MEK and p-ERK levels.

We then set to explore the relationship between Slo and dRaf expressions. An antibody against the fragment of dRaf from aa 331 to 466 was generated in Guinea pigs (Fig. 3d). The dRaf^GOF^ protein contained a truncated sequence with a smaller molecular weight of 39 kDa as compared with the full length dRaf of 88 kDa (Fig. 3d) [57]. Our immunofluorescent staining results showed that the strong dRaf signal co-localized with glial specific GFP in the cytoplasm of *dRaf*^*GOF*^ glioma cells (Fig. 3e). Western blot data confirmed that dRaf^GOF^ was highly expressed in glioma brains, while the expression levels of the full length *wt* dRaf was low (Fig. 3f). Furthermore, insufficiency of *slo* was accompanied by the downregulation of dRaf^GOF^ levels (Fig. 3f, g), while the levels of full length dRaf (88 kDa) remained unchanged (Fig. 3f, g). It seemed that only truncated dRaf^GOF^ levels had fallen upon *slo* knockdown. Since the expression of dRaf^GOF^ was driven by *repo*-GAL4 and the total mRNA levels of *dRaf* showed no significant changes (Fig. 3h), the downregulation of the dRaf^GOF^ was likely due to its degradation. This leaves it unclear as to why the full length dRaf levels remain unchanged. One possibility is that the full length dRaf is more stable than the truncated form.

We further asked whether overexpression of *slo* would upregulate dRaf expression in the *wt* brain, leading to tumor-like phenotypes. A *Drosophila* line with *slo* overexpression driven by *repo*-Gal4 in *wt* background was used in our study. Raised expression levels of Slo were confirmed by RT-PCR and Western blot (Fig. S5a, S5b). Slo overexpression resulted in not only larger brain sizes but also increased numbers of proliferating glial cells as compared with controls (Fig. S5c–S5e). Remarkably, dRaf expression was also upregulated, without any significant changes in RNA levels (Fig. S5a, S5b). As expected, the p-MEK levels were also elevated, suggesting that over-expression of Slo promoted the glial cell proliferation via the BRAF/MAPK pathway. More importantly, it showed that elevated Slo alone was able to upregulate dRaf levels, which in turn gave rise to tumor like phenotypes. In summary, Slo protein level is elevated in *dRaf*^*GOF*^ glioma cells. Knockdown of *slo* not only downregulates dRaf^GOF^ expression but also ameliorates tumor phenotypes. Overexpression of Slo elevates dRaf expression in *wt* glial cells. Thus, expressions of Slo and dRaf^GOF^ are co-dependent and mutually regulated.

### *KCNMA1* expression is elevated in human glioma with* BRAF*^*V600E*^ mutation

**Fig. 4:**
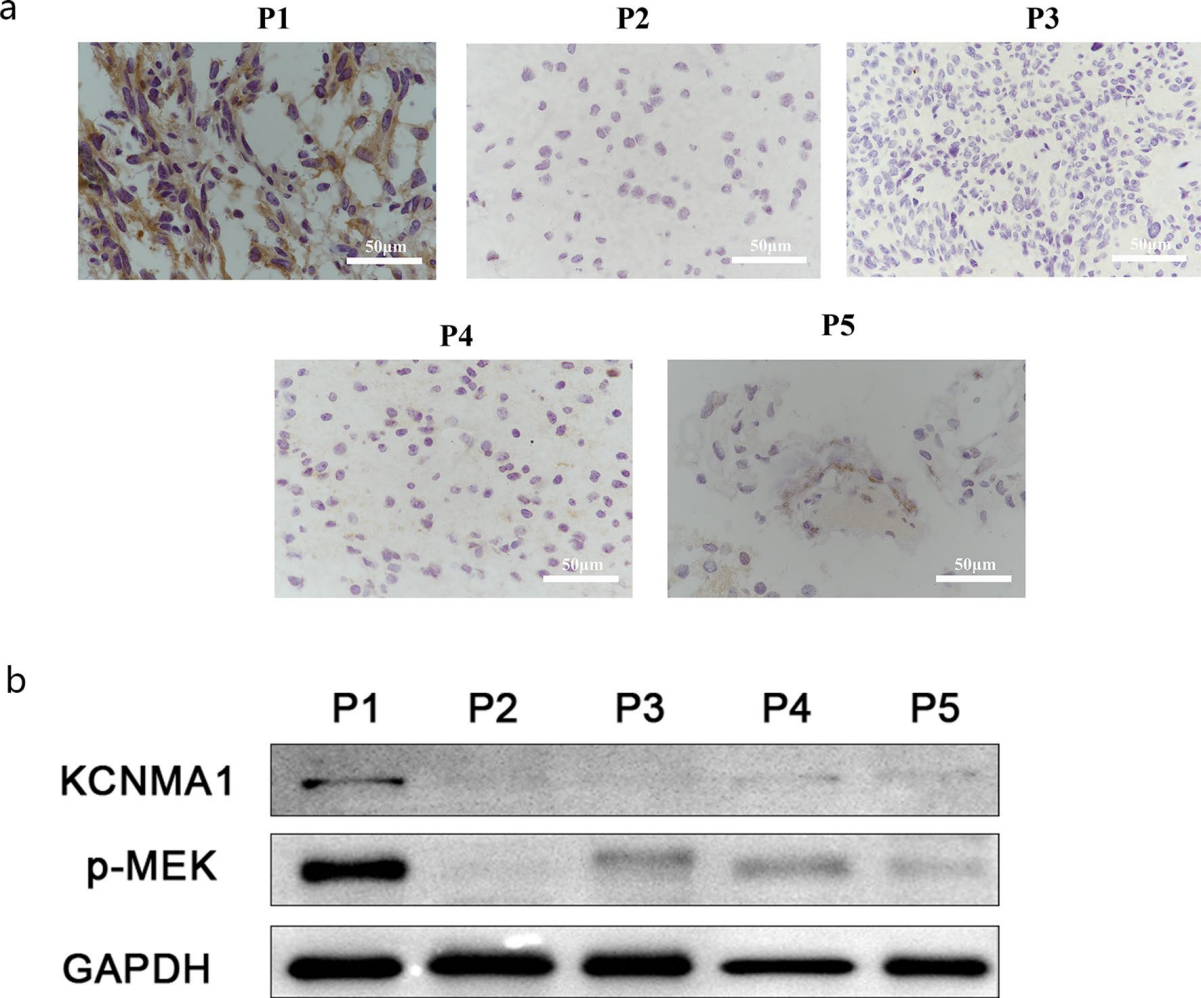
The expression of KCNMA1 was raised in glioma patient with *BRAF*^*V600E*^. **a** Immunohistochemistry (IHC) estimation of KCNMA1 protein levels. Sample P1 exhibited obvious KCNMA1 protein expression (dark brown) in five pediatric glioma samples. **b** Western blot result showing KCNMA1 and p-MEK levels of the five glioma samples. GAPDH serves as internal reference

Having established that Slo functions on *dRaf*^*GOF*^ glioma progression in *Drosophila* larval brains, it is important to move on to consider if the same mechanism is represented in human patients. Since human *BRAF* mutations have been often detected in children, five glioma samples, classified as WHO grade I–V types, were collected from pediatric patients. Clinical data of these samples were obtained from the medical records (Table 1). We first estimated the expression of human ortholog of *slo, KCNMA1,* using a commercial anti-KCNMA1 antibody. The sample P1, which was diagnosed as a hair cell astrocytoma and classified as a WHO grade I glioma, exhibited high levels of KCNMA1 expression while signals from the other four samples were either low or undetected (Fig. 4a).

**Table 1:** Information on glioma samples from Children's Hospital, Zhejiang University School of Medicine

Characteristics			Pathological analysis		*BRAF* Mutation detection
Sample ID	Age	Gender	Pathological diagnosis	Pathologic stage	
P1	4y2m	F	Hair cell astrocytoma	WHO I	BRAF V600E 21.33%
P2	3y4m	F	Mixed neuronal glioma	WHO I	NO
P3	6y9m	F	Glioblastoma	WHO III–IV	NA
P4	2y9m	M	Anaplastic glioma	WHO III	NO
P5	4y5m	F	Hair cell myxoid astroglioma	WHO I	NO

Five glioma samples from the Children’s Hospital, Zhejiang University School of Medicine. The information includes Sample ID, age, gender, pathological diagnosis, pathological stage and *BRAF* mutation detection

*y* years, *m* month, *F* female, *M* male, *NO* no *BRAF* mutation, *NA* not available

To validate the raised expression of *KCNMA1* in this sample, Western Blot analysis was performed. As expected, the expression of *KCNMA1* was highest in sample P1 (Fig. 4b), which was consistent with the immunochemical results. Remarkably, DNA sequencing data showed that the sample P1 carried the *BRAF*^*V600E*^, a well-studied and constitutively active form of BRAF mutation. No *BRAF* mutations were identified in any of the other four samples (Table 1). We then examined the status of BRAF/MAPK pathway. The extremely high level of p-MEK was reflected the hyper-activated status of the BRAF/MAPK pathway in sample P1 (Fig. 4b). These observations followed our findings in *Drosophila* that constitutively activated *BRAF*^*V600E*^ promoting the ion channel *KCNMA1* expression in glioma cells.

### Lack of KCNMA1 suppresses the proliferation and MAPK pathway in *BRAF*^*V600E*^ glioma cells

**Fig. 5:**
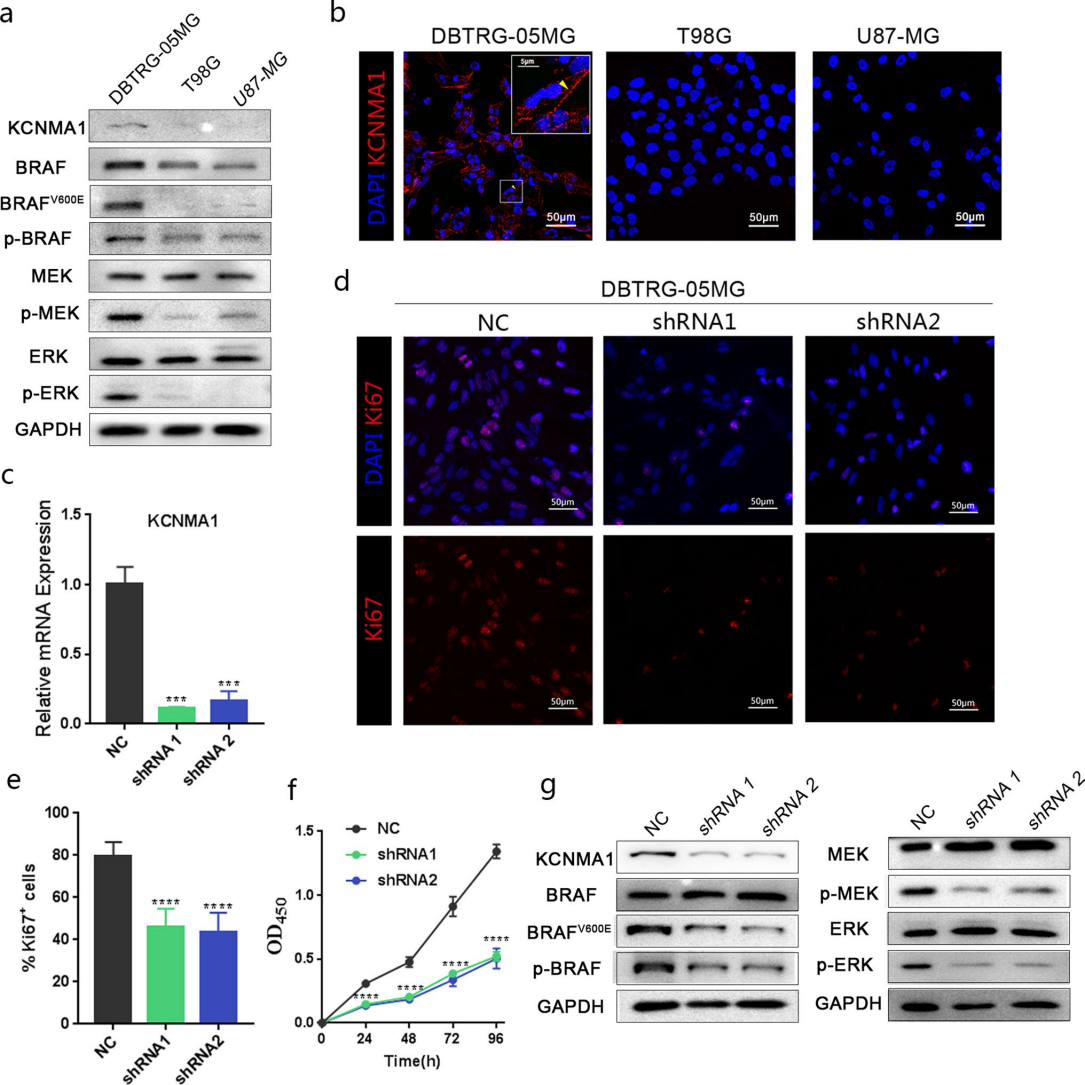
KCNMA1 suppression inhibits the proliferation and MAPK pathway in *BRAF*^*V600E*^ glioma cells. **a** Western blot analyses of three glioblastoma cell lines (DBTRG-05MG, T98G and U87-MG) showing that only in DBTRG-05MG (*BRAF*^*V600E*^) the expression of KCNMA1 was detected with p-BRAF levels, as well as p-MEK and p-ERK, elevated. **b** Confocal images of cells from three glioblastoma cell lines with only DBTRG-05MG (*BRAF*^*V600E*^) showing anti-KCNMA1 staining (red and DAPI blue). **c** Quantitative RT-PCR results indicating the efficiency of *KCNMA1* RNAi knockdown. The data are plotted as mean ± SD. ***P < 0.001. **d** Estimation of cell proliferation of DBTRG-05MG cells with anti-Ki67 staining (red and DAPI, blue). The numbers of Ki67 positive cells had decreased significantly in the presence of *KCNMA1* shRNAs. **e** Proliferation was suppressed by *KCNMA1* shRNAs. The data are plotted as mean ± SD. ****P < 0.0001. **f** A CCK-8 assay was used to examine the proliferation rate of DBTRG-05MG cells. The absorbance at 450 nm was measured at 0, 24, 48, 72 and 96 h (****P < 0.0001). **g** Western blot analysis of DBTRG-05MG cells showed that the expression of KCNMA1 was decreased and p-BRAF levels, as well as p-MEK and p-ERK, were also downregulated in the presence of *KCNMA1* shRNAs. GAPDH served as internal reference in all Western blot works

To study *KCNMA1* function required for human glioma progression, the glioblastoma cell line DBTRG-05MG, a heterozygous mutant carrying *BRAF*^*V600E*^, was employed in our study together with other two glioblastoma cell lines (U87-MG and T98G) with *wt* BRAF as controls. U87-MG carries mutations in *NF1* and *PTEN*, while T98G carries the mutations in *PTEN* and *TP53* (cellosaurus.org). The phosphorylation at serine 729 (S729) of BRAF (p-BRAF) was used as a marker of activated BRAF [58, 59]. Western Blot with a specific anti-p-BRAF showed that the level of p-BRAF was significantly higher in DBTRG-05MG cells than that in two control glioma cell lines (Fig. 5a). It was most likely that the protein configuration of the *BRAF*^*V600E*^ mutation facilitated BRAF phosphorylation [60]. DBTRG-05MG also showed very high levels of p-MEK and p-ERK, indicating the hyperactive status of BRAF/MAPK pathway (Fig. 5a). Importantly, as judged by the Western blot and immunofluorescence staining, only the DBTRG-05MG cell line showed elevated KCNMA1 expression (Fig. 5a, b). KCNMA1 in mammalian cell lines was also membrane-bound, correlating to what had been previously observed in *Drosophila* (Fig. 5b).

To investigate the potential roles of *KCNMA1* in the glioma cell line with *BRAF*^*V600E*^ mutation, two shRNA targeting non-overlapping regions of *KCNMA1* transcript were individually cloned into lentivirus derived vectors and introduced into the DBTRG-05MG cell line. The knockdown efficiency of these two shRNAs was confirmed by RT-PCR and Western blot (Fig. 5c, g). Ki67, a well-known proliferation marker was employed in this study. These two shRNAs showed impeded KCNMA1 expression and significantly reduced glioma cell proliferation in the DBTRG-05MG cell line as compared with the controls (Fig. 5d–f).

In order to uncover the relationship between BRAF/MAPK activation and elevated KCNMA1 expression, we examined BRAF/MAPK components using Western Blot analyses in the DBTRG-05MG cell line. Notably in the presence of RNAi-mediated knockdown of *KCNMA1*, marked reductions of BRAF^V600E^ and p-BRAF were observed (Fig. 5g), as well as significant decreases of p-MEK and p-ERK, while total MEK and ERK remained unchanged. These results indicate that a sufficient quantity of KCNMA1 is required to maintain BRAF^V600E^ and p-BRAF levels (Fig. 5g). This is reminiscent of the Slo function in *Drosophila Raf*^*GOF*^ glioma cells. Taken together, our data indicates that KCNMA1 expression is upregulated in human *BRAF*^*V600E*^ glioma cells. Knockdown of KCNMA1 leads to lower levels of p-BRAF (p-BRAF^V600E^ included), resulting in both down-regulation of cell proliferation and suppression of the BRAF/MAPK pathway. The co-dependency and mutual regulation of KCNMA1 and p-BRAF levels seems to be therefore confirmed for *BRAF*^*V600E*^ glioma cells in a human context.

### Mutual regulation of p-BRAF and KCNMA1 levels requires channel functions

**Fig. 6:**
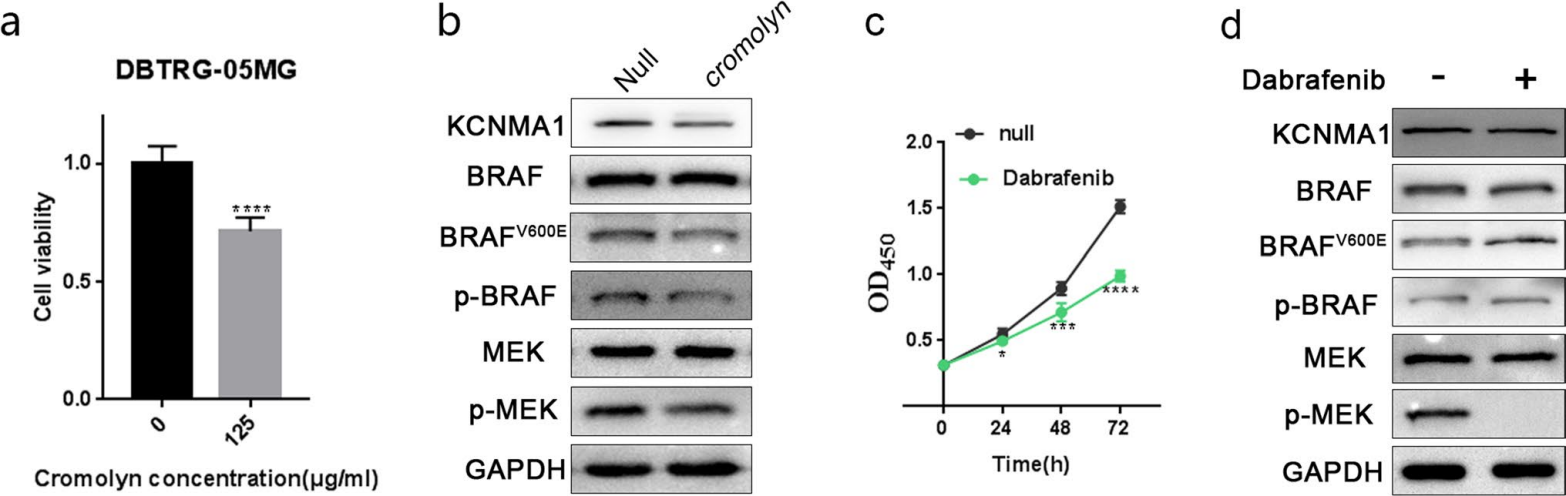
Cromolyn and Dabrafenib treatments in DBTRG-05MG cells. **a** Examination of the cell viability of DBTRG-05MG cells with or without cromolyn treatment (125 μg/ml) by CCK-8 assay at 48 h (****P < 0.0001). **b** Western blot analysis of DBTRG-05MG cells with cromolyn treatment (125 μg/ml) showing levels of KCNMA1 as visibly downregulated together with a slight decrease of BRAF^V600E^, p-MEK and p-ERK. **c** Dabrafenib (1 nM) inhibited the proliferation rates of DBTRG-05MG cells when CCK-8 assay was used to measure the absorbance at 0, 24, 48 and 72 h (*P < 0.05. *** P < 0.001. **** P < 0.0001). **d** Western blot analysis of DBTRG-05MG cell with Dabrafenib (1 nM) treatment showing that the only p-MEK levels were suppressed while p-BRAF and KCNMA1 levels remained unchanged. GAPDH served as internal reference for all Western blots

To understand the mechanism of the mutual regulation of p-BRAF and KCNMA1 levels, we addressed the question whether the KCNMA1 K^+^ channel function was involved in this process. KCNMA1 encodes the pore-forming alpha subunit of the MaxiK channels that mediate the export of K^+^. Such channels are activated by membrane depolarization and an increase in cytosolic Ca^2+^ [22, 23]. Cromolyn sodium, an FDA-approved anti-allergy drug involving in calcium ion transport [61, 62], was predicted as a potential inhibitor for KCNMA1[63]. Correspondingly, we observed decreased cell viability in DBTRG-05MG cells upon cromolyn treatment (Fig. 6a), and unambiguous reductions of p-BRAF, BRAF^V600E^ and p-MEK were detected in the presence of cromolyn inhibition (Fig. 6b). It is interesting to note that blockage of K^+^ channel activity also leads to lower levels of KCNMA1, which could be due to decreased levels of p-BRAF. The result from cromolyn treatment demonstrates the co-dependency of p-BRAF and KCNMA1 levels and confirms that the K^+^ channel function is involved in this process.

In the glioma cell line, p-BRAF activated downstream signaling pathways accompanied by higher KCNMA1 expression (Fig. 5a). However, it remained unclear if the increased KCNMA1 expression depended on the activation of the entire pathway. Therefore, we employed Dabrafenib [64], a selective inhibitor blocking MEK phosphorylation by mutated forms of BRAF kinase in our study. Cell growth assays showed that Dabrafenib significantly suppressed the proliferation of DBTRG-05MG cells (Fig. 6c). Under this condition, the high levels of p-BRAF and KCNMA1 remained unchanged and no p-MEK signal was undetected (Fig. 6d). Thus, we concluded that the mutual regulations of p-BRAF and KCNMA1 were not dependent on p-MEK.

### p-BRAF and KCNMA1 levels are linked to membrane depolarization

**Fig. 7:**
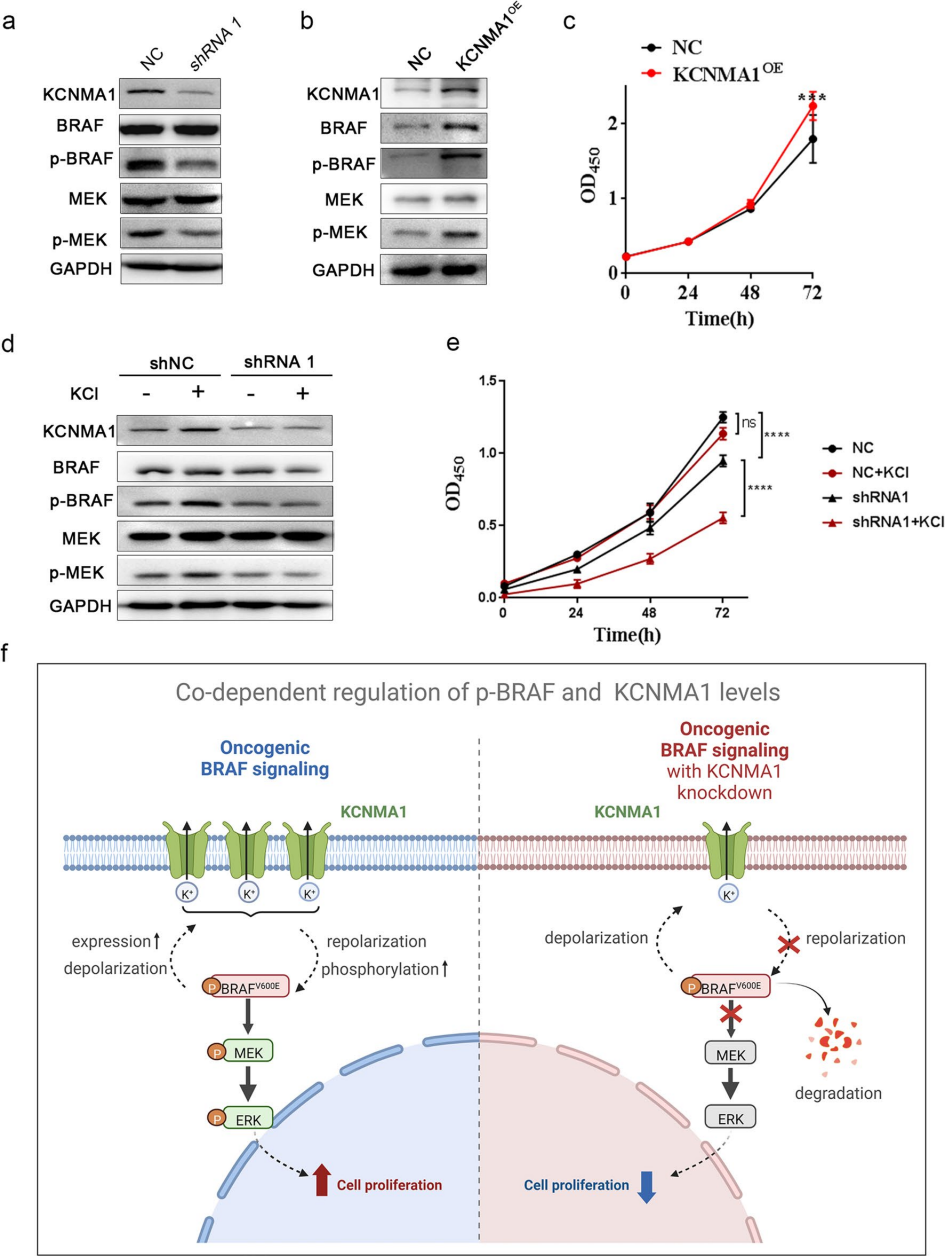
The mechanisms of co-dependency of p-BRAF and KCNMA1. **a** Western blot analysis of HEK293T cell showing moderate levels of KCNMA1 and p-BRAF, and other BRAF/MAPK pathway components. shRNA1 transfections suppressed not only KCNMA1 but also p-BRAF and p-MEK. **b** Western blot analysis indicating that KCNMA1 overexpression (KCNMA1^OE^) in HEK293T cells leads to not only increased KCNMA1 expression, but also higher levels of BRAF, p-BRAF and p-MEK. **c** The proliferation rates of HEK293T cells with KCNMA1 overexpression (KCNMA1^OE^) were examined with CCK-8 assay (***P < 0.001). **d** In the presence of 20 mM KCl in the culture medium, levels of KCNMA1, p-BRAF and p-MEK were raised slightly. The effects of 20 mM KCl were completely blocked in the presence of *KCNMA1* shRNA1. **e** Summary of HEK293T cells proliferation rates of control, shRNA1 treated, and subsequent treatments with or without 20 mM KCl with CCK-8 assay (ns, not significant. ****P < 0.0001). **f** Diagram depicts the proposed mechanism of the co-dependent regulation of p-BRAF and KCNMA1 levels. In gliomas, the oncogenic *BRAF* mutations lead to membrane depolarization and elevated KCNMA1 expression. Raised KCNMA1 repolarizes the membrane potential and sustains p-BRAF levels. This then promotes tumor cell proliferation (left). When *KCNMA1* is knocked down, insufficient amount of KCNMA1 is then available to repolarize the membrane potential. Thus, cell growth is inhibited and p-BRAF^V600E^ is degraded (right)

We then wanted to know whether any co-dependency of p-BRAF and KCNMA1 levels also occurs in cells with *wt* BRAF. In the human embryonic kidney cell line HEK293T (*wt* BRAF), basal levels of p-BRAF and KCNMA1 were detectable (Fig. 7a). p-BRAF was then noted as clearly downregulated upon *KCNMA1* knockdown, while the total expressions of BRAF remained unchanged (Fig. 7a) Concurrent p-MEK decreases were also observed, confirming decreased p-BRAF activity (Fig. 7a). We also raised KCNMA1 expression to see whether p-BRAF levels would follow. Indeed, the overexpression of KCNMA1 also resulted in elevated levels of p-BRAF, BRAF expression, and a corresponding increase in cell proliferation (Fig. 7b, c). These observations confirmed the co-dependency of p-BRAF and KCNMA1 levels in HEK293T cells.

It has been reported that the addition of KCl in cell culture medium depolarizes the membrane potential and activates the BRAF/MAPK pathway [65, 66]. HEK293T cells were therefore treated with 20 mM KCl to induce membrane depolarization. As expected, p-BRAF levels were upregulated (Fig. 7d) and, interestingly, the KCNMA1 protein levels were also increased (Fig. 7d). We note that the depolarization of membrane also activates KCNMA1 K^+^ channels [22, 23]. We then wanted to know whether the KCNMA1 was required for the activation of BRAF/MAPK pathway by depolarization. Knockdown of *KCNMA1* in the presence of 20 mM KCl failed to elevate p-BRAF or p-MEK (Fig. 7d). This result suggests that the KCNMA1 channel is necessary for the activation of the BRAF/MAPK pathway upon membrane depolarization induced by KCl.

We further explored the physiological significance of the co-dependency of p-BRAF and KCNMA1 regulation in HEK293T cells. The cultured cells were treated with 20 mM KCl, with untreated cells as control. Cell growth was then monitored. In the presence of KCl alone, the proliferation of HEK293T cells was almost unaffected, while with additional knockdown of *KCNMA1,* cell proliferation was dramatically suppressed (Fig. 7e). These results uncover the robust nature of the codependency of p-BRAF and KCNMA1 levels and suggest that both are regulated by membrane potential changes. In summary, p-BRAF levels show strong positive correlation with depolarization status. It is KCNMA1, as a K^+^ channel, that functions to provide compensatory repolarization of the membrane which is required to maintain optimal cell growth conditions.

## Discussion

In this study, we have focused on the dependency between glioma growth and ion channel function. The *Drosophila Raf*^*GOF*^ tumor model mimics gliomas that carries *BRAF* mutations [38] and show corresponding phenotypes such as glial cell over-proliferation, seizure-like behavior, and shortened life span. Using the *Drosophila Raf*^*GOF*^ glioma model, together with patient samples and cell lines, we have uncovered the mechanisms of how K^+^ channels *slo/KCNMA1* and p-BRAF strongly enhance glioma growth.

KCNMA1 has been noted as highly expressed in glioblastomas (high grade gliomas). Inconsistent conclusions have been drawn linking the KCNMA1 levels with glioblastoma malignancy [67–69]. Such inconsistencies could be due to the different genetic backgrounds of these gliomas. Based on our investigation, we have concluded that KCNMA1 expression is not simply biased toward high grade gliomas, but is linked more specifically to the BRAF mutations that enhance tumor proliferation. It would be interesting to look at more samples from patients in future study to strengthen this observation.

The trans-membrane protein Slo exhibits a low expression in the *wt Drosophila* brain. We have shown that dRaf^GOF^ promotes Slo expression and that the knockdown of *slo* brings down dRaf^GOF^ in *dRaf*^*GOF*^ gliomas. In addition, the overexpression of Slo in *wt* background also leads to an elevated dRaf level, resulting in BRAF/MAPK pathway activation. The glioblastoma cell line DBTRG-05MG with the *BRAF*^*V600E*^ mutation [70, 71] showed high levels of both p-BRAF and KCNMA1 with a correspondingly hyper-activated BRAF/MAPK pathway. The co-dependency of p-BRAF and KCNMA1 levels was therefore confirmed for this cell line. The same co-dependency of p-BRAF and KCNMA1 levels was then also recognized in the HEK293T cell line, which was derived from non-tumor tissue with *wt BRAF*. This p-BRAF and KCNMA1 level codependency in mammalian cells was highly reminiscent of that previously observed in *Drosophila Raf*^*GOF*^ glioma brains. Thus, we believe that the co-dependency of dRaf^GOF^/p-BRAF and Slo/KCNMA1 is a conserved phenomenon occurring in many different cells and across various species.

It is known that the DBTRG-05MG cells are heterozygous for *BRAF*^V600E^ and contain a copy of *wt* BRAF [70, 71]. BRAF^V600E^ is highly phosphorylated as compared with its *wt* copy[6], which again resembles that observed in the *Drosophila Raf*^*GOF*^ glioma model [38, 72]. In particular, we noticed that the in *slo* or *KCNMA1* knockdown experiments, dRaf^GOF^ or BRAF^V600E^ levels decreased obviously while the endogenous *wt* counterparts remained unchanged. Therefore, it is likely that the p-dRaf^GOF^ or p-BRAF^V600E^ is more susceptible for protein degradation. When the channel protein is knocked down, it is p-dRaf^GOF^ or p-BRAF^V600E^, rather than their *wt* counterparts that degrade quickly.

Two inhibitor experiments have uncovered interesting features of mutual regulations of p-BRAF and KCNMA1 levels in DBTRG-05MG cells. We found that the p-BRAF and KCNMA1 levels remained unaffected even when MEK phosphorylation was inhibited by Dabrafenib [64]. Thus we propose that an unknown mechanism exists to relay the signals from p-BRAF to establish the KCNMA1 levels in glioma cells, and that this occurs without the involvement of p-MEK. Furthermore, in the presence of cromolyn, K^+^ channel activity was decreased [63], leading to lower levels of p-BRAF and diminished glioma cell viability. This result indicates that the K^+^ channel function is involved in the mutual regulation of p-BRAF and KCNMA1 levels and suggests that the KCNMA1 channel could be a potential drug target specific for tumors that are induced by BRAF mutations.

The study here on HEK293T cells cultured with KCl provides a further clue why p-BRAF and KCNMA1 levels are codependent. Treatment of KCl leads to membrane depolarization [65, 66], which in turn activates KCNMA1 channel activity [22, 23]. The fact that cells are able to cope with these membrane potential changes in the presence of KCNMA1 may be due to KCNMA1 being able to compensate for depolarization by promoting repolarization of the membrane potential [25]. Correspondingly, poor cell growth was then observed when such repolarization is inhibited by *KCNMA1* knockdown in these depolarized cells. Based on this, we propose that the depolarization by *BRAF* mutations [20] may be an unhealthy condition for cell growth and that KCNMA1 acts to counter this and repolarize the membrane potential to re-optimize conditions for these cells. In this scenario, there must be a membrane potential-mediated mechanism that co-regulates p-BRAF and KCNMA1 levels for optimal membrane potential. In this relationship, high levels of p-BRAF will therefore also require elevated expressions of KCNMA1, and vice versa. However, the exact nature of this membrane potential-mediated feedback loop remains to be elucidated.

In conclusion, our study reveals that p-BRAF and KCNMA1 levels are mutually regulated and co-dependent (Fig. 7f). In glioma cells, high levels of p-BRAF^V600E^ leads to membrane depolarization and elevated KCNMA1 expression. Raised KCNMA1 then acts to repolarize the membrane potential and sustain p-BRAF levels, which promotes tumor cell proliferation. When *KCNMA1* is knocked down, insufficient amounts of KCNMA1 are available to repolarize the membrane potential. Cell growth is therefore inhibited and p-BRAF^V600E^ becomes degraded. Therefore, the co-dependency of KCNMA1 and p-BRAF is highlighted as critical for BRAF-mutated glioma progression and, due to this, KCNMA1 could be a valuable potential therapeutic drug target for the treatment of tumors with *BRAF* mutations.

## Supplementary Information

Below is the link to the electronic supplementary material.

Supplementary file1 (DOCX 4014 KB)

## Data Availability

Data sharing is not applicable to this article as no datasets were generated or analyzed during the current study. The manuscript has data included as electronic supplementary material.

## References

[1] Wan PT (2004). Mechanism of activation of the RAF-ERK signaling pathway by oncogenic mutations of B-RAF. Cell.

[2] Davies H (2002). Mutations of the BRAF gene in human cancer. Nature.

[3] Roskoski R (2010). RAF protein-serine/threonine kinases: structure and regulation. Biochem Biophys Res Commun.

[4] De Luca A (2012). The RAS/RAF/MEK/ERK and the PI3K/AKT signalling pathways: role in cancer pathogenesis and implications for therapeutic approaches. Expert Opin Ther Targets.

[5] Schubbert S, Shannon K, Bollag G (2007). Hyperactive Ras in developmental disorders and cancer. Nat Rev Cancer.

[6] Lavoie H, Therrien M (2015). Regulation of RAF protein kinases in ERK signalling. Nat Rev Mol Cell Biol.

[7] Samatar AA, Poulikakos PI (2014). Targeting RAS-ERK signalling in cancer: promises and challenges. Nat Rev Drug Discov.

[8] Louis DN, The, (2021). WHO classification of tumors of the central nervous system: a summary. Neuro Oncol.

[9] Collins VP, Jones DT, Giannini C (2015). Pilocytic astrocytoma: pathology, molecular mechanisms and markers. Acta Neuropathol.

[10] Jones DT (2008). Tandem duplication producing a novel oncogenic BRAF fusion gene defines the majority of pilocytic astrocytomas. Cancer Res.

[11] Schindler G (2011). Analysis of BRAF V600E mutation in 1,320 nervous system tumors reveals high mutation frequencies in pleomorphic xanthoastrocytoma, ganglioglioma and extra-cerebellar pilocytic astrocytoma. Acta Neuropathol.

[12] Dougherty MJ (2010). Activating mutations in BRAF characterize a spectrum of pediatric low-grade gliomas. Neuro Oncol.

[13] Lassaletta A (2017). Therapeutic and prognostic implications of BRAF V600E in pediatric low-grade gliomas. J Clin Oncol.

[14] Lim CS (2017). BRaf signaling principles unveiled by large-scale human mutation analysis with a rapid lentivirus-based gene replacement method. Genes Dev.

[15] Yeh E (2018). Patient-derived iPSCs show premature neural differentiation and neuron type-specific phenotypes relevant to neurodevelopment. Mol Psychiatry.

[16] Gasser A (2010). Two Nedd4-binding motifs underlie modulation of sodium channel Nav1.6 by p38 MAPK. J Biol Chem.

[17] Wittmack EK (2005). Voltage-gated sodium channel Nav1.6 is modulated by p38 mitogen-activated protein kinase. J Neurosci.

[18] Poolos NP, Bullis JB, Roth MK (2006). Modulation of h-channels in hippocampal pyramidal neurons by p38 mitogen-activated protein kinase. J Neurosci.

[19] Koh HY (2018). BRAF somatic mutation contributes to intrinsic epileptogenicity in pediatric brain tumors. Nat Med.

[20] Goz RU, Akgül G, LoTurco JJ (2020). BRAFV600E expression in neural progenitors results in a hyperexcitable phenotype in neocortical pyramidal neurons. J Neurophysiol.

[21] Pallanck L, Ganetzky B (1994). Cloning and characterization of human and mouse homologs of the Drosophila calcium-activated potassium channel gene, slowpoke. Hum Mol Genet.

[22] Magleby KL (2003). Gating mechanism of BK (Slo1) channels: so near, yet so far. J Gen Physiol.

[23] Horrigan FT, Aldrich RW (2002). Coupling between voltage sensor activation, Ca^2+^ binding and channel opening in large conductance (BK) potassium channels. J Gen Physiol.

[24] Robitaille R, Charlton MP (1992). Presynaptic calcium signals and transmitter release are modulated by calcium-activated potassium channels. J Neurosci.

[25] Petersen OH, Maruyama Y (1984). Calcium-activated potassium channels and their role in secretion. Nature.

[26] Basile MS (2019). KCNMA1 expression is downregulated in colorectal cancer via epigenetic mechanisms. Cancers (Basel).

[27] Ma G (2017). KCNMA1 cooperating with PTK2 is a novel tumor suppressor in gastric cancer and is associated with disease outcome. Mol Cancer.

[28] Ramírez A (2018). Calcium-activated potassium channels as potential early markers of human cervical cancer. Oncol Lett.

[29] Oeggerli M (2012). Role of KCNMA1 in breast cancer. PLoS ONE.

[30] Khaitan D (2009). Role of KCNMA1 gene in breast cancer invasion and metastasis to brain. BMC Cancer.

[31] Mound A (2013). Molecular interaction and functional coupling between type 3 inositol 1,4,5-trisphosphate receptor and BKCa channel stimulate breast cancer cell proliferation. Eur J Cancer.

[32] Atkinson NS, Robertson GA, Ganetzky B (1991). A component of calcium-activated potassium channels encoded by the Drosophila slo locus. Science.

[33] Singh S, Wu CF (1989). Complete separation of four potassium currents in Drosophila. Neuron.

[34] Elkins T, Ganetzky B, Wu CF (1986). A Drosophila mutation that eliminates a calcium-dependent potassium current. Proc Natl Acad Sci USA.

[35] Gribkoff VK, Starrett JE, Dworetzky SI (2001). Maxi-K potassium channels: form, function, and modulation of a class of endogenous regulators of intracellular calcium. Neuroscientist.

[36] Fernández MP (2007). Impaired clock output by altered connectivity in the circadian network. Proc Natl Acad Sci USA.

[37] Read RD (2011). Drosophila melanogaster as a model system for human brain cancers. Glia.

[38] Penman CL (2015). Current understanding of BRAF alterations in diagnosis, prognosis, and therapeutic targeting in pediatric low-grade gliomas. Front Oncol.

[39] Sang R (2022). Mxc, a Drosophila homolog of mental retardation-associated gene NPAT, maintains neural stem cell fate. Cell Biosci.

[40] Wu D (2019). RanGAP-mediated nucleocytoplasmic transport of Prospero regulates neural stem cell lifespan in Drosophila larval central brain. Aging Cell.

[41] Alfonso TB, Jones BW (2002). gcm2 promotes glial cell differentiation and is required with glial cells missing for macrophage development in Drosophila. Dev Biol.

[42] Elkins T, Ganetzky B, Wu C-F (1986). A gene affecting a calcium-dependent potassium current in Drosophila. Proc Natl Acad Sci.

[43] Elkins T, Ganetzky B (1988). The roles of potassium currents in Drosophila flight muscles. J Neurosci.

[44] Mulcahy Levy JM, McMahon M (2018). Linking brain tumors and epileptic seizures. Nat Med.

[45] Xing H (2021). Clinical characteristics of BRAF V600E gene mutation in patients of epilepsy-associated brain tumor: a meta-analysis. J Mol Neurosci.

[46] Ganetzky B, Wu CF (1982). Indirect suppression involving behavioral mutants with altered nerve excitability in DROSOPHILA MELANOGASTER. Genetics.

[47] Kasbekar DP, Nelson JC, Hall LM (1987). Enhancer of seizure: a new genetic locus in Drosophila melanogaster defined by interactions with temperature-sensitive paralytic mutations. Genetics.

[48] Pavlidis P, Tanouye MA (1995). Seizures and failures in the giant fiber pathway of Drosophila bang-sensitive paralytic mutants. J Neurosci.

[49] Burg MG, Wu CF (2012). Mechanical and temperature stressor-induced seizure-and-paralysis behaviors in Drosophila bang-sensitive mutants. J Neurogenet.

[50] Sailer CA (2006). Immunolocalization of BK channels in hippocampal pyramidal neurons. Eur J Neurosci.

[51] Contet C (2016). BK channels in the central nervous system. Int Rev Neurobiol.

[52] Awasaki T (2008). Organization and postembryonic development of glial cells in the adult central brain of Drosophila. J Neurosci.

[53] Stork T (2008). Organization and function of the blood–brain barrier in Drosophila. J Neurosci.

[54] Whitfield ML (2006). Common markers of proliferation. Nat Rev Cancer.

[55] López-Sáez JF (1998). Cell proliferation and cancer. Histol Histopathol.

[56] Feitelson MA (2015). Sustained proliferation in cancer: mechanisms and novel therapeutic targets. Semin Cancer Biol.

[57] Melnick MB, Perkins LA, Lee M, Ambrosio L, Perrimon N (1993). Developmental and molecular characterization of mutations in the Drosophila-rafserine/threonine protein kinase. Development.

[58] Haling JR (2014). Structure of the BRAF-MEK complex reveals a kinase activity independent role for BRAF in MAPK signaling. Cancer Cell.

[59] Vido MJ (2018). BRAF splice variant resistance to RAF inhibitor requires enhanced MEK association. Cell Rep.

[60] Cope N (2018). Mechanism of BRAF activation through biochemical characterization of the recombinant full-length protein. ChemBioChem.

[61] Shapiro GG, König P (1985). Cromolyn sodium: a review. Pharmacotherapy.

[62] Corcia A (1986). Characterization of the ion channel activity in planar bilayers containing IgE-Fc epsilon receptor and the cromolyn-binding protein. Embo j.

[63] Rask-Andersen M, Masuram S, Schiöth HB (2014). The druggable genome: Evaluation of drug targets in clinical trials suggests major shifts in molecular class and indication. Annu Rev Pharmacol Toxicol.

[64] Roskoski R (2018). Targeting oncogenic Raf protein-serine/threonine kinases in human cancers. Pharmacol Res.

[65] Zubkov AY, Rollins KS, Zhang JH (2002). KCl activates mitogen-activated protein kinase in rabbit bailar artery. Biochem Biophys Res Commun.

[66] Shim JH (2015). KCl mediates K(+) channel-activated mitogen-activated protein kinases signaling in wound healing. Arch Plast Surg.

[67] Weaver AK, Bomben VC, Sontheimer H (2006). Expression and function of calcium-activated potassium channels in human glioma cells. Glia.

[68] Khaitan D, Ningaraj N (2019). Evidence of calcium-activated potassium channel subunit alpha-1 as a key promoter of glioma growth and tumorigenicity. Glioma.

[69] Catacuzzeno L (2015). Reconciling the discrepancies on the involvement of large-conductance Ca(2+)-activated K channels in glioblastoma cell migration. Front Cell Neurosci.

[70] Quentmeier H (2001). Immunocytochemical analysis of cell lines derived from solid tumors. J Histochem Cytochem.

[71] Bignell GR (2010). Signatures of mutation and selection in the cancer genome. Nature.

[72] Xia F (2008). Raf activation is regulated by tyrosine 510 phosphorylation in Drosophila. PLoS Biol.

