# Supplementary material for: Co-dependent regulation of p-BRAF and potassium channel KCNMA1 levels drives glioma progression
Source: Cell Mol Life Sci. 2023 Feb 10;80(3):61. doi: 10.1007/s00018-023-04708-9 (PMC9918570; doi:10.1007/s00018-023-04708-9)
Supplement: Supplementary file 1 — Supplementary file1 (DOCX 4014 KB) [file 18_2023_4708_MOESM1_ESM.docx]

# Supplementary information

Co-dependent regulation of p-BRAF and potassium channel KCNMA1 levels drives glioma progression

Fig. S1


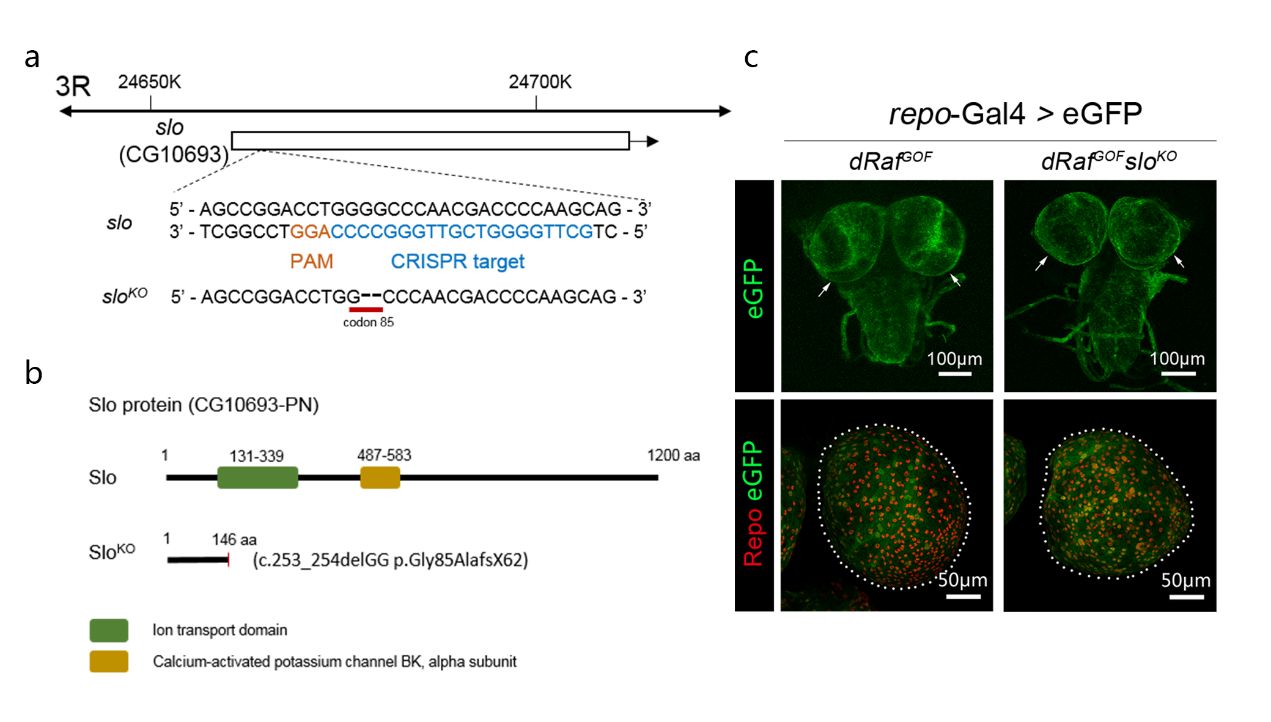


**Fig. S1** **Enlarged brain lobes of *dRaf^GOF^* glioma were rescued by *slo^KO^***

(a) *slo^KO^* mutant generation with CRISPR/Cas9 method. sgRNA sequences are indicated in blue and PAM sequences in orange. The red line marks the missing 2bp bases in codon 85. (b) Schematic presentation of the full-length Slo and Slo^KO^ proteins. (c) Confocal images of the third-instar larval brain lobes of *dRaf^GOF^* glioma and *dRaf^GOF^* glioma with *slo^KO^* (anti-Repo, red and eGFP, green). The enlarged brain lobes (arrows) of *dRaf^GOF^* gliomas were rescued by *slo^KO^*. Both *dRaf^GOF^* and eGFP were driven by *repo*-Gal4.

Fig. S2


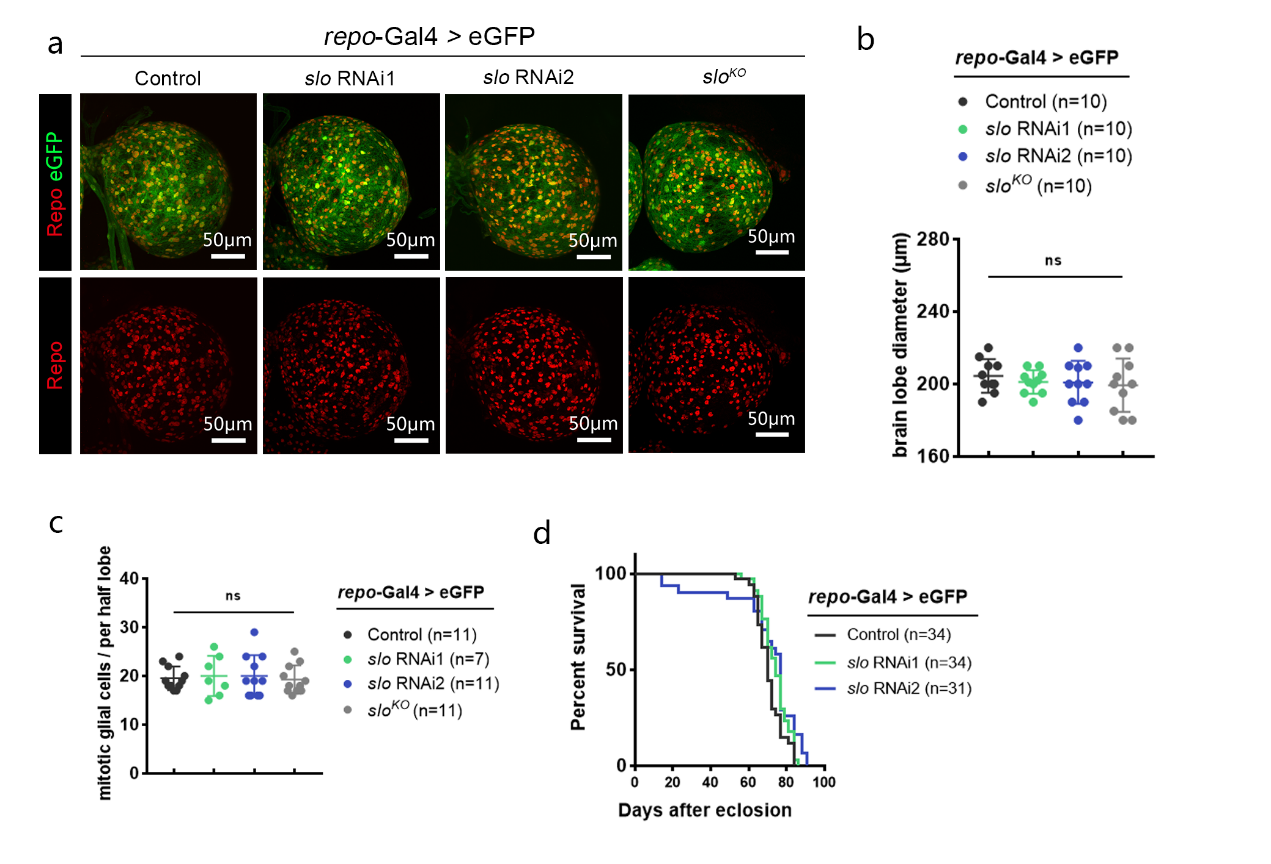


**Fig. S****2 Lack of Slo in glial cells does not affect brain development**

(a) Confocal images of the third-instar larval brain lobes of the control and of the two *slo*-RNAi as driven by *repo*-Gal4 and *slo^KO^* animals (anti-Repo, red and eGFP, green). (b) Statistical analysis of the diameter of the third-instar larval brain lobes of the control, two *slo*-RNAi treated, and *slo^KO^* animals. The data are plotted as mean± SD. ns, not significant. n = 10. (c) Statistical quantifications of the mitotic glia cell numbers for each half lobe of the control, two *slo*-RNAi treated and *slo^KO^* samples. The data are plotted as mean± SD. ns, not significant. (d) Survival rates of the control and two *slo*-RNAi treated animals.

Fig. S3


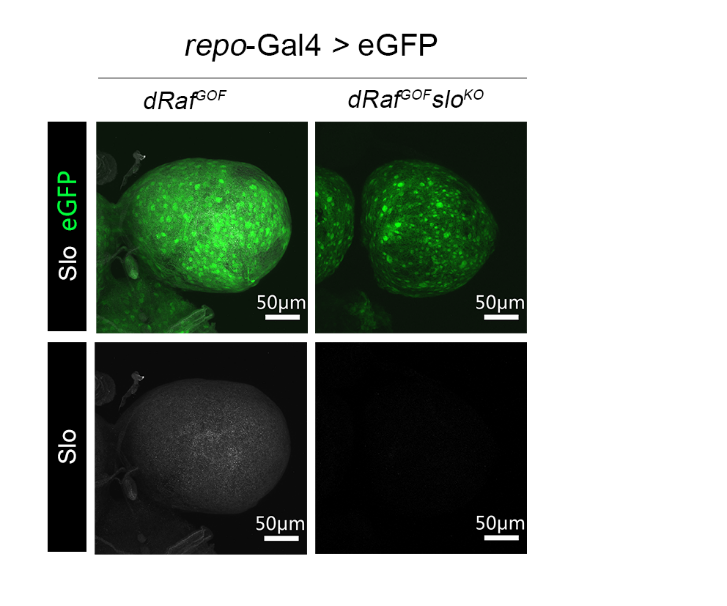


**Fig. S3 Slo is undetectable in *dRaf^GOF^* glioma with *slo*^KO^**

Confocal images of the third-instar larval brains of *dRaf^GOF^* glioma and *dRaf^GOF^* glioma with *slo*^KO^ (anti-Slo, white and eGFP, green).

Fig. S4


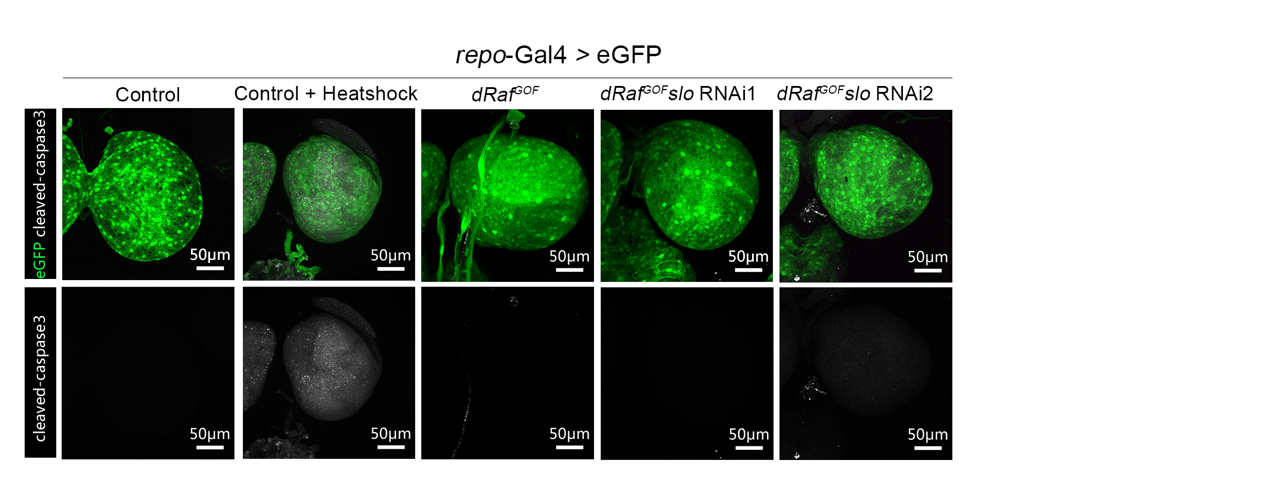


**Fig. S4 Inhibition of glioma progression by *slo* knockdown occurs independent of apoptosis**

Confocal images of the third-instar larval brain lobes of the control, *dRaf^GOF^* gliomas, and two *dRaf^GOF^* gliomas with *slo*-RNAi treatment (anti-cleaved caspase3, white and eGFP, green). No significant anti- cleaved caspase3 signals were detected. A larval brain lobe with heatshock as positive control.

Fig. S5


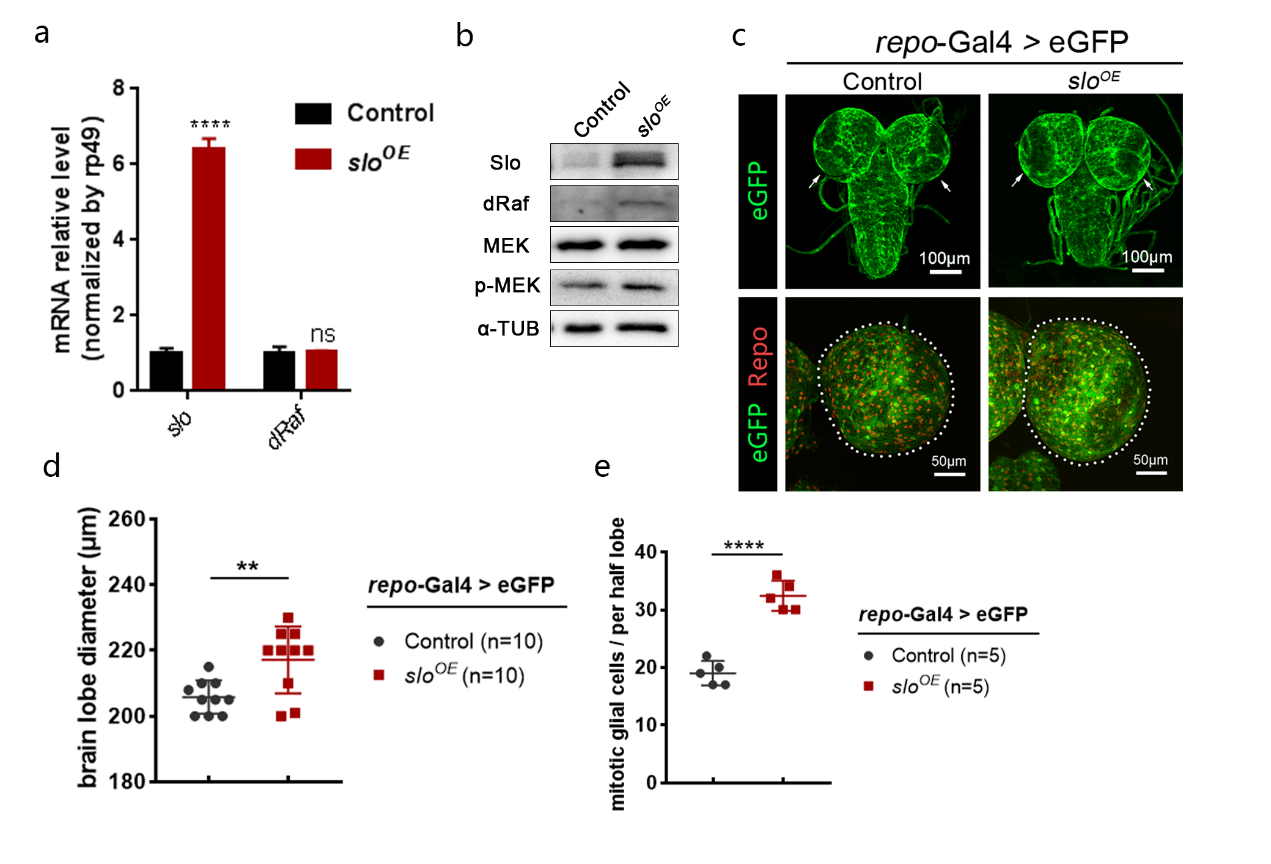


**Fig. S5 *slo^OE^* elevates dRaf levels and promotes cell proliferation**

(a) Quantitative RT-PCR data of *slo* and *dRaf* mRNA levels of the control and *slo* overexpression lines (*slo^OE^*). *dRaf* mRNA levels were not affected by *slo* overexpression. *rp49* as internal reference (****P<0.0001. ns, not significant). (b) Slo overexpression led to higher levels of dRaf and p-MEK. α-tubulin (A-TUB) as internal reference. (c) Confocal images of the third-instar larval brain lobes of the control and *slo^OE^* (anti-Repo, red and eGFP, green). (d) Statistical quantification of the larval brain lobe sizes of the control and *slo^OE^* (**P<0.01; n = 10). (e) Quantifications of mitotic glial cell numbers for each half lobe of the control and *slo^OE^* (****P<0.0001; n=5).
